# Evaluating Drug Deposition Patterns from Turbuhaler® in Healthy and Diseased Lung Models of Preschool Children

**Published:** 2022

**Authors:** Ravishekar Kannan, Ryan Arey, Andrzej Przekwas, Ariel Berlinski, Narender Singh

**Affiliations:** 1 CFD Research Corporation, 701 McMillian Way NW, Suite D, Huntsville, Alabama 35806, USA; 2 Pediatric Aerosol Research Laboratory, Arkansas Children’s Research Institute. 13 Children’s Way, Little Rock, AR 72202, USA

**Keywords:** Quasi-3D, Q3D, pediatric, Turbuhaler®, 3-year-old, Preschool Children, Peripheral:Central ratio, P:C ratio, Quasi-3D whole lung model, diseased subjects

## Abstract

The efficacy of pediatric oral drug delivery using dry powder inhalers, such as Turbuhaler®, is dependent on the age and health of the test subjects. The available clinical data for these studies is scant and rarely provide correlations between the health condition and the regional lung deposition. In particular, the data and the correlations for pre-school children are minimal. Deposition simulations were performed using the newly developed Quasi-3D whole lung model to analyze the effect of health conditions on the regional lung deposition from the Turbuhaler® in 3-year-old children. The healthy lung model was created from CT scan data. Cystic-fibrosis models were created by uniformly constricting the airways to various degrees. The simulated drug deposition outcomes were validated against the available experimental data. The results show that, while the dose deposited in the lungs exhibits minor variations, the Peripheral:Central (P/C) ratio is strongly affected by both the health condition and the inflow variations. The above ratio is reduced by ~30% for the severely diseased case, compared to its healthy counterpart, for the same inhalation profile. This indicates that lower doses reach the peripheral lung, in pediatric cystic-fibrosis subjects, thus requiring a larger therapeutic dose.

## Introduction

The multidose dry powder inhaler (DPI) Turbuhaler^®^, has been widely used for the treatment of adults with respiratory diseases. Radiolabeled Scintigraphic studies have shown that Turbuhaler^®^ is capable of delivering ~25% of the packaged dose into the lung, in adults [1-4]. In particular, several research studies indicate that the Turbuhaler^®^ delivers a greater fraction of the packaged dose into the lungs than other DPIs and metered dose inhalers (MDIs) [3-10]. One compelling reason for the above is attributed to the peak inspiratory flow rate (PIFR) associated with the Turbuhaler^®^ [2,8,11-15]. A higher PIFR corresponds to a greater fine particle fraction (FPF) of the active particle ingredient (API) [2,13,16], thus allowing a larger lung deposition fraction, especially in the lower and peripheral lungs.

Since the PIFR of young children is significantly lower than that of adults, the FPF of the API delivered in children is lower than that of adults [17]. In theory, this partially negates the efficacy of Turbuhaler^®^, when used in children. Nevertheless, there are a handful of studies that have shown the efficacious clinical outcomes of using the Turbuhaler^®^ for preschool children, younger than 4 years [18,19]. However, there are several issues associated with these studies: (i) They are few in number, hence variations are hardly reported, (ii) They are mainly for diseased pediatric subjects (due to healthy children not being experimented on for ethical reasons). Hence there are no comparisons between diseased and healthy subjects, (iii) Due to the paucity of such studies, the effect of drug depositions as a function of the disease progression has not been analyzed, and (iv) neither has been enough subjects, to obtain the variations in the regional drug deposition metrics, such as, penetration index which is a measure of peripheral to central deposition ratio (P/C ratio).

The motivation behind this study is to provide computationally based answers to all of the issues mentioned in the last paragraph. Thus, in the presented study, we used the recently developed Quasi-3D (Q3D) whole lung framework [20], to obtain the regional lung depositions (budesonide drug) using Turbuhaler^®^, in 3-year old children. The healthy lung model was created from retrospectively obtained CT scan data. Three variants of the drug delivery in healthy subjects were simulated by using the three experimentally measured Particle size distributions (PSDs). Four cystic fibrosis lung geometries were created by uniformly and progressively reducing airway caliber in order to represent different degrees of lung health. The overall lung deposition matched well with the available experimental data. The P:C ratios were computed for all three cases and noticeable variations were observed. In particular, this P:C ratio is about ~16-30% lower in the moderate-severely diseased lung models than the healthy lung models. These analyses demonstrate that much lower doses reach the peripheral lung in pediatric cystic-fibrosis subjects (for the same inflow conditions), thus requiring a larger therapeutic dose (~16-30% higher for the moderate-severely diseased case). In conclusion, the use of pediatric whole lung models, both healthy and diseased, can be used to explore several what-if scenarios on the regional lung deposition, which arise from the negative features reported in the earlier paragraph.

## Materials and Methods

### The Quasi-3D (Q3D) whole lung model

CFD Research (CFDRC) has performed the Q3D lung deposition using the dimensions corresponding to the 3-year-old male subject. The geometry data (STL file) was provided by Dr. Paul Segars, Associate Professor of Radiology at Duke University. Most known lung models typically contain the geometry of only the first 6-9/3-4 branch generations at-best/average, respectively. At CFDRC, we have developed a first of its kind - full 24 generation lung model of an adult male human. This work was done in collaboration with the FDA CDER team [39,20]. This adult lung model was created from the zygote STL [40]. In this section, we will describe the process (in the context of pediatric lungs) for (i) using the CT scan imaging data to extend the Q3D lung till the end of the tracheobronchial (TB) limit (i.e., 15 generations), and (ii) constructing “sac-trumpet” like control volumes at the end of the TB exits to mimic the alveoli.

**Figure 1:**
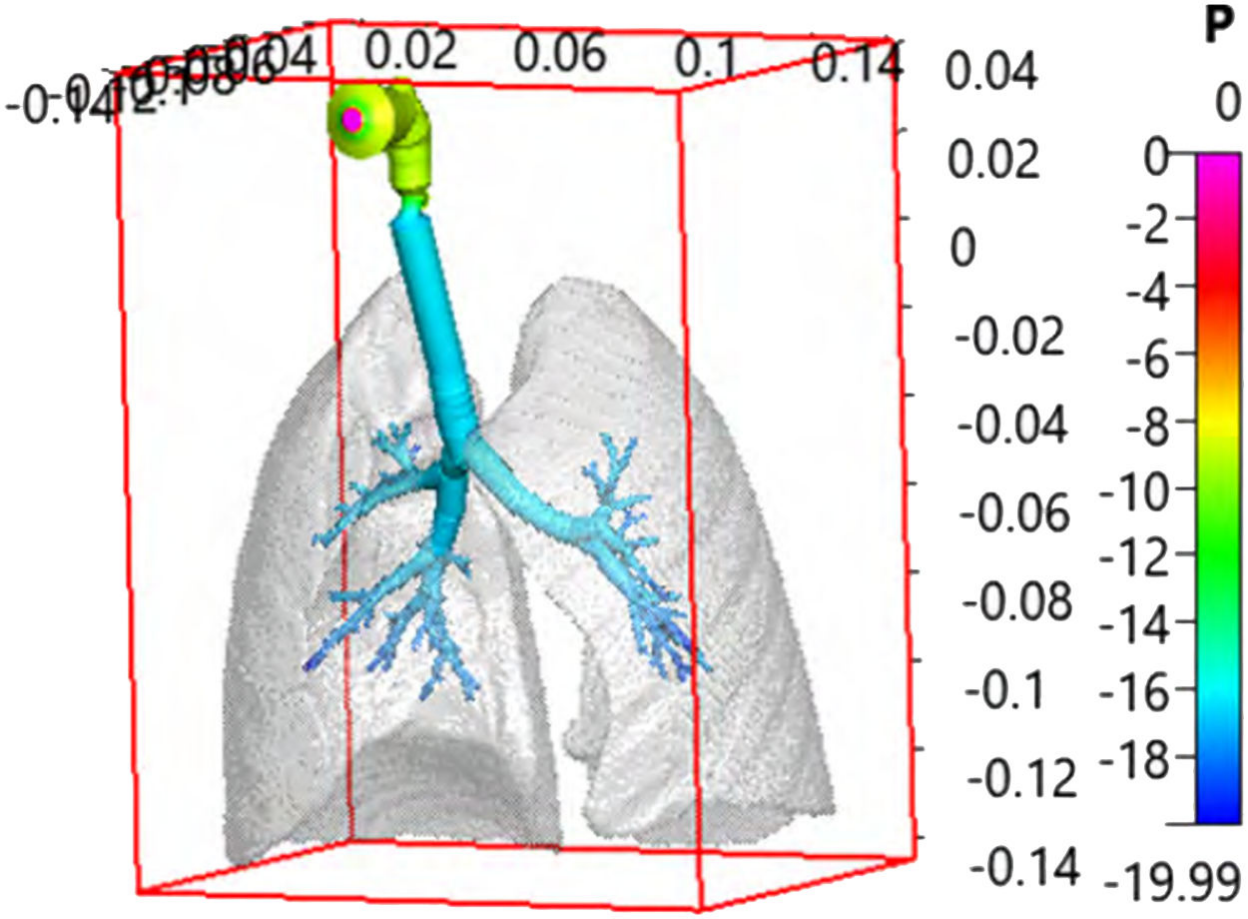
The original Q3D lung of the 3-year subject, enclosed in the lung lobes, is colored by pressure profiles. Inhalation rate=3 L/min. The dimensions presented in the bounding box are in SI units (meters).

As the first step, we extended the truncated Q3D lung to the end of the TB limit. The lung lobes provide the outer boundary, for the extension process. Figure 1, shows the lung lobes, enclosing the original Q3D lung (created from the CT scans) for the three-year old subject.

**Figure 2:**
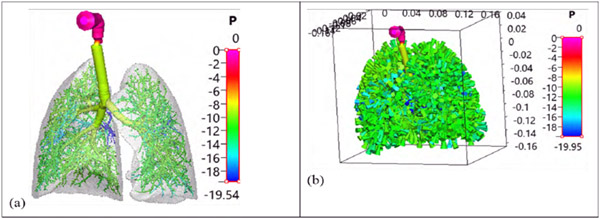
Case (a): THe 3-year-old TB Q3D lung, enclosed in the lung lobes. Case (b): The 3-year-old whole lung (sac-trumpet + TB Q3D) model, colored by pressure profiles (in Pascal) for an inhalation rate of 3 L/min. The dimensions presented in the bounding box are in SI units (meters).

We then (i) adapted the algorithm of Karch, et al. [21] to extend the current Q3D airways, to the end of the TB limit and (ii) implemented sac-trumpet-like control volumes, at each of the TB outlets. Figure 2a shows the lung extended to the TB limit. The final step, is the insertion of alveolar “trumpet-sac” control volumes, at each of the TB outlets. Figure 2b shows the complete Q3D lung, i.e., after the insertion of the trumpet-sac control volumes. This was described in detail in our recently published study for adults [20], including the algorithm to penetrate to the end of the TB limit (determined by the lobe geometry) and the construction of alveolar trumpet-sacs.

The dimensional metrics are as follows: (i) the constructed models have Functional Residual Capacities (FRC) of 581 ml and for the 3-year-old subject; (ii) the constructed models have a TB FRC of 35.1 ml, for the 3-year-old subject. The whole lung Q3D model was generated to match the experimentally observed FRC of 581 ml [22]. The generated lung model has a TB FRC, comparable to the allometrically scaled value from adults (155 ccs * (0.581 L/2.300 L) = 39.15 ccs).

The cystic-fibrosis variants are created by uniformly scaling the radii from the trachea to the alveolus. We created four variants, corresponding to reductions of 2%,4%,6%, and 11.5% in the radius. These correspond to the different levels of the disease progression, with the 11.5% reduction corresponding to the severely diseased state. These simulated radius reductions are within the experimentally observed radius reductions, which are discussed in Section 3.2.

### Inhalation inputs for the model

**Figure 3:**
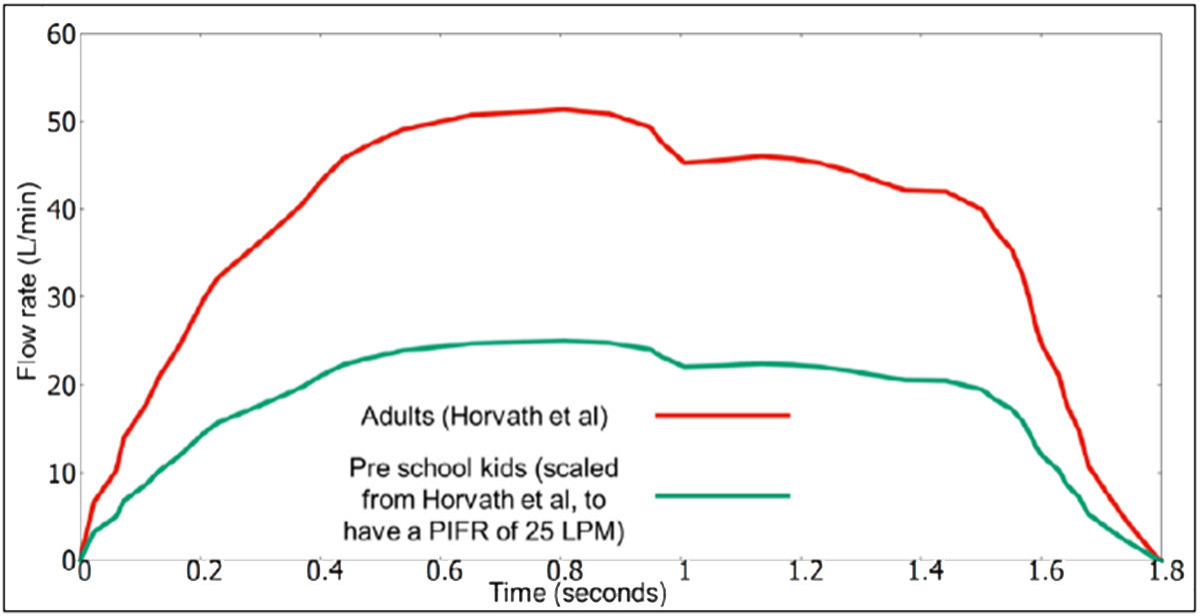
The flowrare used in our simulations, for the turbuhaler^®^ simulations.

The PIFR was set to 25 LPM (liters per minute), as per the experimental measurements of Devadason, et al. [17]. Due to difficulty, and lack of literature data, in obtaining the time-varying flow rate for the Turbuhaler^®^ in young children, we adapted an existing flowrate for adults [23]. The adult flowrate is scaled down, to achieve a PIFR of 25 LPM (to correspond to the 3-year-old subjects [17]). This is provided in Figure 3.

### Particle size distribution (PSD) for the model

Three stage-wise distribution measurements from Turbuhaler^®^ were reported by Devadason, et al. [17]. Table 1 provides these stage-wise dose depositions.

Devadason, et al. [17] used a multistage liquid impinger (MSLI), at an inhalation flowrate of 60 LPM to measure the PSD. Hence, for a given lung model (corresponding to a given health condition), we have a total of 3 PSD-based variations. Thus, using these 3 variations we can analyze the variation for the lung deposition metrics which will make the simulation process more realistic.

NOTE: The PIFR for the Turbuhaler^®^ in preschool children is 25 LPM. This is considerably smaller than the 60 LPM flowrate used to measure the PSDs using the MSL impactor. However, the PSD data for such low flowrates (<= 25 LPM) does not exist and we have to employ the data created using the MSLI impactor at 60 LPM. In reality, the diameters of particles measured at less than 25 LPM are expected to be larger than the ones measured at 60 LPM. Hence the deposition simulations using this available PSD might be under-predicted in the mouth region.

## Results

### Regional lung deposition using the healthy lung model

In this section, we provide the results from the deposition simulations for the healthy subject in terms of deposition fractions, as a proportion of the dose entering the mouth. Table 2 provides the lung deposition fraction, the mouth deposition fraction, and the P:C ratio using the healthy lung model, and a comparison with the experimental measurements of Devadason, et al. [17]. We followed the convention of Devadason, et al. [17], who defined the central region as the lung-domain representing half the width of the lung and one-third the lung-height. The remaining section was classified as the peripheral region. In the context of modelling, the region from the trachea (denoted as generation 0) to the 7^th^ generation, matched the definition of Devadason, et al. [17]. The main observations are as follows:

1. Only two subjects (age group: 3-5 years) took part in Devadason’s study. Hence the reported data has few variations in the lung deposition.
2. The simulated healthy lung model predicts the lung deposition fraction, which is very similar to the experimental measurement.
3. Inspite of using 3 variants, we get a variation of just ~2% between the maximum and the minimum reported total lung deposition fraction.
4. However, we observed significant variations in P:C ratio obtained using the simulation. This implies that changes in the PSD result in significant changes in the deposition pattern in the lung, due to the complex diffusion, inertial, and sedimentation deposition patterns.
5. Even though our simulations correspond to the healthy subject, the computed depositions are not far off from the experimental data which was obtained for diseased subjects. However, these can be improved by conducting diseased state (cystic-fibrosis) simulations, as discussed in the next subsection.
6. In particular, the mouth deposition is slightly underpredicted by the model. This is probably due to using the PSDs corresponding to the 60 LPM, as discussed in the earlier paragraph.

NOTE: The basic assumption is that the drug transport is governed by the air transport (inhalation and exhalation). Hence, as per this computational model, the P:C ratio will not be influenced just by changing the doses (for instance between 1000 micrograms or 250 micrograms).

**Figure 4:**
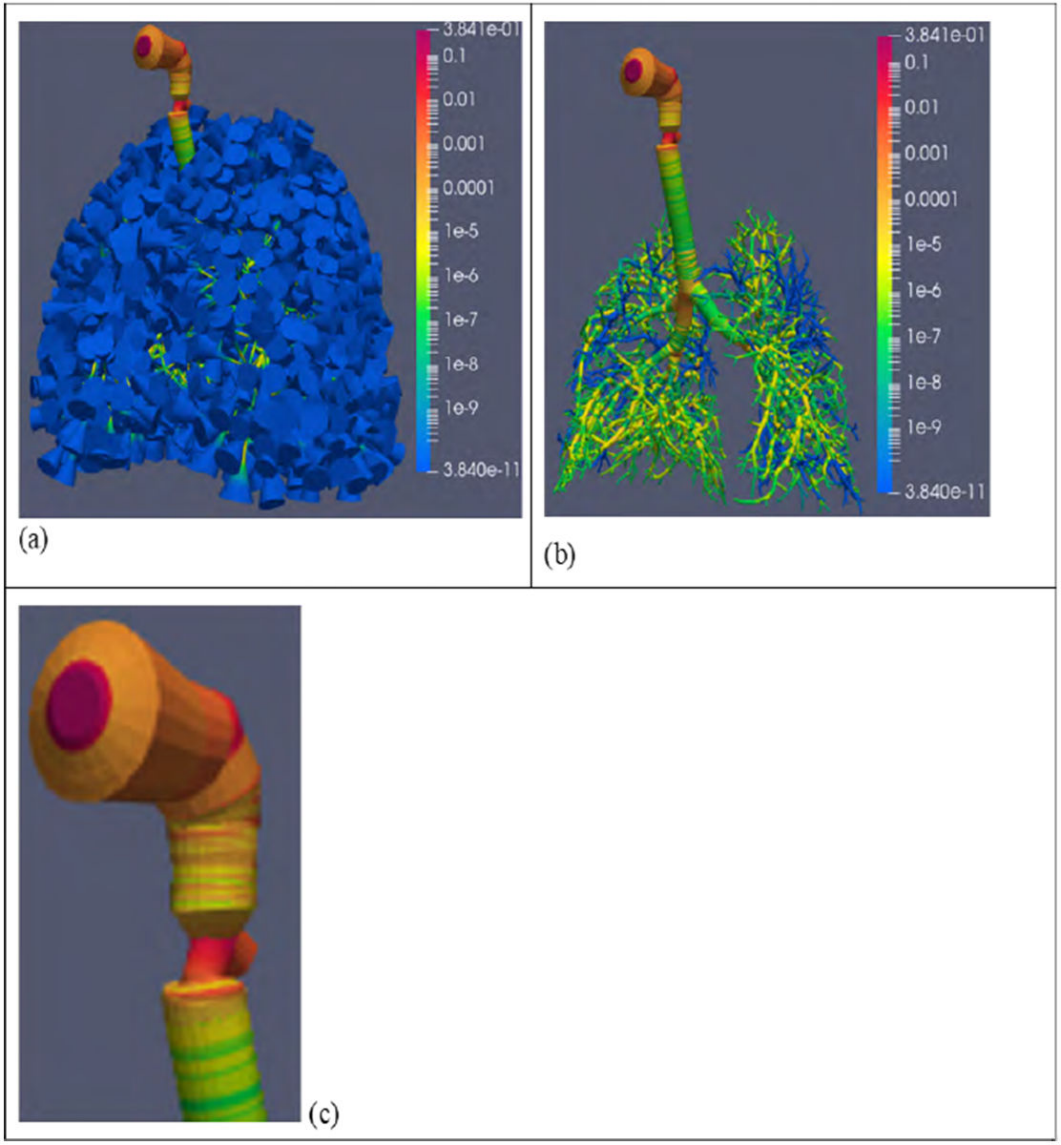
Depositions in the healthy lung model. The mass entering the mouth is normalized to 1.0. Case (a): The depositions in the whole lung; Case (b): The depositions displayed, after blanking the respiratory trumpet-sacs; Case (c): A zoom-in of the larynx region.

Figure 4a shows the complete Q3D lung, colored by the deposited drug fractions. The drug entering the mouth is normalized to 1.0. As expected, there is a significant fraction of the drug deposited in the mouth region. However, we observe a sizeable fraction deposited in the larynx region (~15%), from Figure 4b. Such a large fraction concentrated in the larynx suggests the occurrence of local side effects like the hoarseness of the voice (dysphonia), which happens due to the action of the steroid inhaler on the larynx [41]. We also observe that even though the P:C ratio is more than 1, the drug hardly reaches the alveolus (generation 24). However, it does reach the lower lung generations, as shown in Figure 4b.

### Regional lung deposition using the diseased (cystic-fibrosis) lung models

In this section, we provide the results from the deposition simulations for the cystic-fibrosis diseased lung models. Four different states of disease progression are presented. They correspond to a diameter reduction of 2% (mild), 4% (medium) and 6% (moderate), and 11.5% (severe). Scant literature information is available to support this disease progression reduction in humans. The only available information is from the porcine studies (the lobar and bronchial anatomy of pigs is similar to that of humans [24]). Airway lumen areas in newborn piglets with cystic-fibrosis, were reported to be approximately 50% lower than their healthy counterparts [25]. This corresponds to a radius reduction of ~29% in cystic-fibrosis affected subjects. In a related study, the trachea of infants (i.e. humans) less than two weeks of age, were analyzed: the mean area, for the cystic-fibrosis affected and the healthy subjects were 7.6 mm*mm and 9.7 mm*mm respectively [26]. This corresponds to a radius reduction of ~11.5% in cystic-fibrosis affected subjects. Hence, we varied the levels of disease progression, from from 2 to 11.5% constrictions in the diameters.

NOTE: We have used a uniform radius reduction in this study. In reality, the reduction might be heterogenous, and the region-specific constriction fractions, can be obtained using imaging data. However, this is beyond the scope of this paper.

We will continue to compare the deposition fraction, as a proportion of the dose deposited in the body. Table 3 presents the deposited drug fraction, for the healthy, diseased models, and from experimental measurements.

The main observations are as follows:

1. As expected, the total lung deposition fraction is nearly unchanged irrespective of the health condition of the lung. Minor variations in the lung deposition (for different health conditions) are observed, due to the diffusion effects. In theory, a lower tracheal diameter (due to constriction: in the diseased lung models) implies a larger concentration (due to mass conservation), and this results in a higher diffusion flux from the trachea to the larynx. However, as expected, this effect is minor.
2. The P:C ratio progressively decreases, as the lung health condition deteriorates.
3. The P:C ratio is about 16-30% lower in the moderate-severely diseased lung. This implies lesser dose reaching the deep lung in the severely diseased subjects.
4. As a consequence, higher therapeutic doses need to be prescribed (compared to healthy and milder cases) for moderate and severely diseased subjects, assuming similar breathing conditions.
5. However, severely diseased subjects might also have a lower inhaled flowrate (including the PIFR), thereby a lower fine particle fraction (due to lower impact collisions in the inhaler), which will further reduce the P:C ratio.
  - This implies that the actual P:C ratios for the severely diseased subjects might be even lower than these model predictions.
6. The variation in the P:C ratio (for a given health condition) is almost the same, irrespective of the health condition of the lung

## Discussions and Conclusions

This study provides the first high-fidelity simulation-based information on the dose delivered to the lungs of 3-year-old children, when using Turbuhaler^®^. The motivation for this study is to fill the gaps associated with the experimental data collection for preschool children, younger than 4 years. These gaps include: (i) Extremely few cases have been experimentally analyzed, thereby resulting in almost-no variations, (ii) No healthy subjects are generally analyzed: thus researchers cannot compare the deposition metrics between healthy and diseased children, (iii) Similarly, correlations between the drug deposition and the varying disease progression levels can’t be determined, and (iv) neither has been enough subjects, to obtain the variations in the regional drug deposition metrics like the penetration index (P:C ratio): thus it is difficult to predict in advance the therapeutic doses required for diseased pediatric subjects.

Using our recently published Q3D whole lung model, we were able to obtain the regional lung depositions (budesonide drug) using Turbuhaler^®^, in 3-year-old children, for various cystic-fibrosis disease progression stages. The healthy lung model was created from retrospectively obtained CT scan data. The cystic-fibrosis lung models (corresponds to different levels of disease progression) were created by uniformly constricting the airways. Three inflow-based particle size distribution (PSD) variants were created, for a lung deposition simulation, for every health condition. The overall lung deposition fraction matched well with the available experimental data. Our simulations showed significant variations in the P:C ratios, irrespective of the health condition of the lung, thereby filling up the gaps left by the paucity of the experimental data. The mouth deposition was slightly under-predicted, due to the use of the PSDs that were experimentally measured, using a larger flowrate.

The P:C ratio progressively decreased, as the health condition of the lung deteriorated. The P:C ratio for the moderately/severely diseased lung was ~16/30% lower than its healthy counterpart. Using these simulated data, we can strongly conclude that larger therapeutic doses need to be prescribed for severely diseased pre-school pediatric subjects, than the data obtained by conducting tests on healthy children. This is even without accounting the possibility that severely diseased subjects might have a lower inhaled flowrate (including the PIFR), thereby a lower fine particle fraction, which will further reduce the P:C ratio. While this is a computational based finding, we will look forward to experimentalists confirming this in the future (using radio labelled drugs).

This study has uncovered valuable information relating to the performance of the Turbuhaler^®^ in childhood, using high fidelity simulations. While, these simulations, are specific to the Turbuhaler^®^ device, they point to the issues relating to orally inhaled drug delivery in pre-school kids. We will conduct similar numerical exercises, to predict the performance of other inhalers.

## Figures and Tables

**Table 1:** Stage-wise depositions (percentage), measured by the impactor for Turbuhaler^®^ device. Data discretized from Devadason, et al. [17].

Stage	Distribution-1	Distribution-2	Distribution-3
Mouth	30.44	21.40	25.93
Stage-1	22.76	27.29	19.52
Stage-2	3.358	7.112	5.813
Stage-3	10.89	14.04	14.90
Stage-4	34.77	32.40	35.84

**Table 2:** Mouth and lung deposition percentages using the Turbuhaler^®^. These depositions are expressed as a percentage of the total body dose. Simulations are conducted using the healthy lung model.

Case	Mouth (%)	Lung(%)	P:C
Devadason et al [17]	86.6 +/−1.7	13.4 +/−1.7	1.3 +/− 0.1
Healthy lung model (3 variants)	80.4-81.2	18.8-19.6	1.40-1.55

**Table 3:** Mouth and lung deposition percentages using the Turbuhaler^®^. These depositions are expressed as a percentage of the total body dose. Simulations are conducted using the healthy lung model and the diseased lung models.

Case	Mouth (%)	Lung (%)	P:C
Devadason et al [17]	86.6 +/−1.7	13.4 +/−1.7	1.3 +/− 0.1
Healthy lung model (3 variants)	80.4-81.2	18.8-19.6	1.40-1.55
Mildly Diseased lung model (3 variants)	80.4-81.3	18.7-19.6	1.33-1.47
Medium Diseased lung model (3 variants)	80.5-81.3	18.7-19.5	1.25-1.39
Moderately Diseased lung model (3 variants)	80.6-81.3	18.7-19.4	1.18-1.30
Severely Diseased lung model (3 variants)	80.8-81.5	18.5-19.2	0.975-1.08

## Data Availability

NA, since all the data have been reported in the paper.

## Appendix

### Appendix A: The deposition computations.

The biggest assumptions are (i) the individual monodispersed particles do not affect each other. This assumption allows us to instantiate multiple invocations of the species transport module, corresponding to different particle sizes, (ii) complex 3D phenomena like flow-recirculation should not overtly affect the deposition process. This way, we can plug analytical deposition expressions, in the Euler-Euler Q3D module, in each computational cell.

Since the transport is using a species transport equation, the species value is set to zero, for the cell averaged concentrations. The inlet concentration *C*_*INLET*_ is set to 1.0, during the actuation period. It is set to zero after the actuation period. Thus, the total mass inhaled (for that mono-dispersed particle class) is $M_{INLET}=\int_{0}^{T_{inhale}}C_{INLET}\cdot A_{INLET}U_{INLET}dt$, where *U_INLET_* is the aerosol inlet velocity.

The deposition process occurs on the Q3D walls. The deposition fraction in each computational cell is given by:

$$\frac{\int_{0}^{\infty }C_{ENTRY}.U.\pi R^{2}\;P_{TOTAL}dt}{\int_{0}^{T_{inhale}}C_{INLET}.A_{INLET}U_{INLET}dt}$$

where *P*_*TOTAL*_ is the overall deposition probability, U is the mean particle transport velocity in that computational cell, R is the radius of the airway segment and *C*_*ENTRY*_ is the concentration at which the aerosol enters the computational cell.

We first compute the probability of deposition in each computational Q3D cell. There are three methods of deposition, that can occur. They are described below:

The probability of the diffusion deposition in each Q3D mesh segment, , *P*_*DIFF*_ is obtained from the analytical expressions for the diffusion deposition in a tube by multiple research programs [1-3]. As noted, by Ingham, et al. [3], several terms of the infinite series equation are needed to compute the end concentration. By limiting the expression to the first 5 terms, we obtain a good approximation for the probability of the diffusional deposition:

(1)
PDRIFF=0.819e-7.315x+0.0976e-44.61x+0.0325e-114x+0.0509e-79.31x∕32

(2)
$$\text{where}\;\;X=\frac{\left(Length∕U_{Mean}\right)}{2R_{0}^{2}∕D}$$

L is the length of the airway segment, U is the mean particle transport velocity in that computational cell, R is the radius of the airway segment, and D is the diffusion coefficient of particles in air given by: $D=\frac{k_{B}TC_{corr}}{6\pi \mu r_{p}}$. *k* is the Boltzmann constant, T is the body temperature, r_p_ is the microparticle radius, μ is the air viscosity. Ccorr is the Cunningham slip correction factor [4], and is given by: Ccorr=1+2λ2rp(A1+A2exp(∕λ−A32rp).

For air, A_1_ = 1.257, A_2_ = 0.400, A_3_ = 0.55 [5]. (80 nm) is the mean free path. For r_p_ = 1.1 μm, C_corr_ = 1.086 because λ is much smaller than the microparticle radius r_p_.

The probability of the impaction in a non-bifurcating tube can be obtained from the semi-empirical formulation of Weiden et al [6] and Pui et al [7]:

$$P_{IMPACT}=1-(1+(\frac{Stk}{0.171}{)}^{0.452\frac{Stk}{0.171}+2.242}{)}^{\frac{-2\theta }{\pi }}$$

where ∅ is the angle of curvature of the bend and *stk* is the local Stokes number.

The probability of the deposition (i.e., sedimentation), due to the acceleration due to the gravity is given by [6]:

$$P_{SEDI}=1-exp\left(-\frac{4gC_{corr}\rho r_{p}^{\;\;2}L\;cos\;\varphi }{9\pi \mu RU}\right)$$

where, *ρ* is the particle density, g is the gravity, *φ* is the inclination angle of the airway segment relative to the gravity (0 for the horizontal tube) and *C_corr_* is the Cunningham slip correction factor. The efficiency *P*_*IMPACT*_ due to impaction deposition, in the case of bifurcating airways is given by [8]:

$$P_{IMPACI}=1-\frac{2}{\pi }\;\cos^{-1}(St\cdot \theta )+\frac{1}{\pi }\;\sin\left(2\;\cos^{-1}(St\cdot \theta )\right);St\cdot \theta <1\;P_{IMPACT}=1;St\cdot \theta \geq 1$$

where θ is the branching angle of the airway generation and *St* is the Stokes’ number

The overall deposition probability is given by

$$P_{TOTAL}=1\;\text{-}\;(1\;\text{-}\;P_{DIFF})(1\;\text{-}\;P_{IMPACT})(1\;\text{-}\;p_{SEDI}).$$

This loss term is implemented in the species transport equation (, in CFDRC’s finite volume method CoBi. Additional details on the species or flow transport can be obtained from previous CoBi publications [9-12].

NOTE: While this Q3D method cannot resolve the minute details like the vortices and the recirculation, this is still a practical spatio-temporal accurate method, to obtain the regional depositions, for realistic inhalation profiles which occur over several seconds. While traditional CFD methods are great in resolving the recirculation and vortices, simulating the depositions in a whole-lung CFD model is computationally exhaustive and impossible. Hence this Q3D approach is a practical alternative.

### Appendix B: The transport and deposition equation, for the aerosol species transport

In every computational Q3D cell, the following equations relate the entry, cell average and exit concentration states:

$$\frac{C_{in}+C_{OUT}}{2}=\bar{C};\;C_{OUT}=(1-P)C_{in}$$

The rate of mass lost, due to the deposition in the Q3D cell is given by:

$$M_{LOSS}^{\;\;{}^{\cdot }}=Q(C_{in}-C_{OUT})=QC_{in}(P)=\frac{2PQ\bar{C}}{2-P}\;,$$

where Q is the flowrate. Plugging this into the species transport equation, integrated over the Q3D cell:

$$Vol.\frac{\partial \bar{C}}{\partial t}+\oint (\nabla (u_{i}C)-D_{i}\nabla^{2}(C))dV=-\frac{2PQ\bar{C}}{2-P}$$

### Appendix C: Mesh refinement analysis

The simulations performed using our whole lung Q3D model is mesh independent. Here are some metrics, to showcase the mesh independence. The P:C ratios from the fine (~145 K cells) and the medium meshes (~122 K cells) are provided. The fine mesh has around ~twice the number of computational cells in the TB region, compared to the medium mesh. The number of cells in the respiratory region (gen 16-24) is the same for the medium and the fine meshes, since that region is the last region of the deposition (i.e., the aerosol exiting the TB will eventually deposit in the respiratory region). These numbers demonstrate mesh independence.

**Table:**

Mesh	Median P:C ratio (Healthy subject)
Medium mesh	1.52
Fine mesh	1.48
